# Efficacy of carboplatin plus S-1 for the treatment of non-small cell lung cancer: A protocol for a systematic review of randomized controlled trial

**DOI:** 10.1097/MD.0000000000015099

**Published:** 2019-04-05

**Authors:** Lei Han, Zhou-Xia Wei, Yu-Feng Lv, Ai-Ying Jiang

**Affiliations:** a Department of Respiratory Medicine, The Affiliated Hongqi Hospital of Mudanjiang Medical University, Mudanjiang; b Department of General Medicine, The First Hospital of Jilin University, Changchun, China.

**Keywords:** carboplatin, efficacy, non-small cell lung cancer, S-1, safety

## Abstract

**Background:**

Non-small cell lung cancer (NSCLC) is the most common lung cancer. Numerous clinical studies have reported that the combination of carboplatin and S-1 (CS) can be used to treat NSCLC effectively. However, no systematic review has been conducted to assess its efficacy and safety for NSCLC. This systematic review aims to evaluate the efficacy and safety of CS for treatment of patients with NSCLC.

**Methods:**

This study will retrieve the following electronic databases from inception to the February 1, 2019: Cochrane Library, EMBASE, MEDILINE, CINAHL, AMED, and 4 Chinese databases without any language limitations. This systematic review will include randomized controlled trials (RCTs) and case-control studies for assessing the efficacy and safety of CS for the treatment of NSCLC. Cochrane risk of bias will be used as methodological quality assessment for each qualified study. The RevMan V.5.3 software will be utilized to synthesize the data and conduct the meta-analysis if it is allowed. The data will be pooled by using the random-effects model or fixed-effects model.

**Results:**

The primary outcome is overall response rate. The secondary outcomes are overall survival, progression-free survival, the disease control rate, and any adverse events.

**Conclusion:**

It will provide latest evidence to determine the efficacy and safety of CS for treatment of patients with NSCLC.

**Ethics and dissemination:**

No research ethic approval is needed in this study because this study will not analyze individual patient data. The results are expected to disseminate through peer-reviewed journals.

**Systematic review registration:**

PROSPERO CRD42019124860.

## 1 Introduction

Lung cancer has become one of the most frequent causes of cancer-related death around the world.^[1–3]^ This disorder has broadly classified into 2 subtypes: small cell lung cancers and non-small cell lung cancer (NSCLC).^[4,5]^ Of these, NSCLC accounts for 80% of all lung cancer cases.^[6]^ It has been reported that about 228,150 new cases, and 142, 670 deaths of NSCLC patients are estimated in 2019 in USA.^[7]^ Although most patients have received surgery, there is still about 1% to 2% risk of relapse for each patient annually even after the resection of lung cancer.^[8]^

Chemotherapy has also been reported to treat NSCLC effectively^[9,10]^, especially for carboplatin, S-1, and combination of carboplatin and S-1 (CS).^[11–15]^ Numerous clinical studies have demonstrated that CS can be utilized to treat NSCLC with promising outcome results.^[16–30]^ However, up to the present, no study has systematically investigated the efficacy and safety of CS for the treatment of NSCLC. Thus, in this systematic review, we will firstly assess the efficacy and safety of CS for the treatment of patients with NSCLC.

## 2 Methods

### 2.1 Objective

This systematic review aims to evaluate the efficacy and safety of CS for the treatment of patients with NSCLC.

### 2.2 Study registration

This study has been registered with PROSPERO CRD42019124860. We will report this study following the guideline of Preferred Reporting Items for Systematic Reviews and Meta-Analysis (PRISMA) Protocol statement.^[31]^

### 2.3 Inclusion criteria for study selection

#### 2.3.1 Type of studies

This study will consider randomized controlled trials (RCTs) and case-control studies of CS for NSCLC. However, the other studies will be excluded, such as non-clinical studies, case reports, case series, and cross-over studies.

#### 2.3.2 Type of participants

This study will include patients with clinically diagnosed of NSCLC, regardless of their race, gender, and age. However, patients with other severe diseases will be excluded, such as other organs cancers, severe heart diseases, and any others that may affect the efficacy evaluation in this study.

#### 2.3.3 Type of interventions

Experimental group: any forms of CS will be included, except the combination of CS with other treatments.

Control group: any interventions can be used, except CS.

#### 2.3.4 Type of outcome measurements

The primary outcome includes overall response rate. The secondary outcomes comprise of overall survival, progression-free survival, the disease control rate, and any adverse events.

### 2.4 Search methods for the identification of studies

#### 2.4.1 Electronic searches

The electronic databases of Cochrane Library, EMBASE, MEDLINE, Cumulative Index to Nursing and Allied Health Literature, Allied and Complementary Medicine Database, Chinese Biomedical Literature Database, China National Knowledge Infrastructure, VIP Information, and Wanfang Data will be retrieved from inception to the February 1, 2019 without any language restrictions. The detailed search strategy for Cochrane Library is built and is presented in Table 1. Similar detailed search strategies will also be applied to any other electronic databases.

**Table 1:**
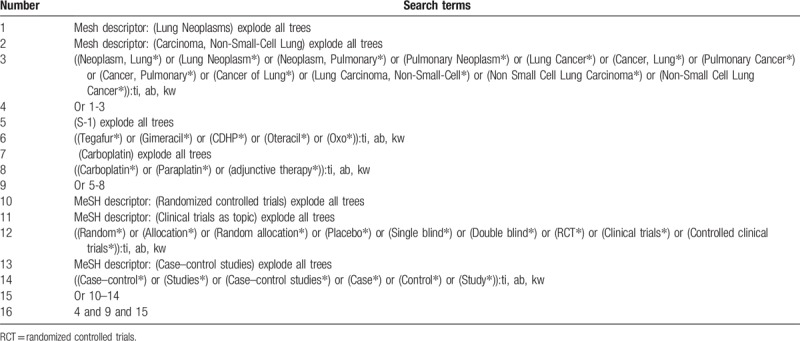
Detailed search strategy for Cochrane Library database.

#### 2.4.2 Other resources

Additionally, clinical registry, Google Scholar, any other related conference proceedings, and reference lists of relevant studies will also be searched in this study.

### 2.5 Data collection and analysis

#### 2.5.1 Selection of studies

The Endnote 7.0 software will be used for study selection by handling the search studies and removing the duplicated studies. Two researchers will independently select all potential studies by screening titles or/and abstracts according to the predefined eligibility criteria. Then, full-texts will also be read carefully if there is insufficient information to help for judgment only by scanning titles or/and abstracts. The whole process of study selection will follow the guidelines of PRISMA and will be presented in PRISMA flow diagram with detailed reasons of exclusion and inclusion at each stage. Any disagreements between the 2 researchers will be resolved by a third researcher.

#### 2.5.2 Data collection and management

Two researchers will independently extract data from each qualified study by using pre-designed data extraction form. Any other divergences will be resolved by consulted a third researcher. All the extracted data will be recorded by Excel 2010. The form of data extraction comprises of the following information:

Study characteristics: title, first author, publication year, and location;
Patient characteristics: race, gender, age, diagnostic criteria, eligibility criteria, number of patients in each group.
Study methods: details of randomization, concealment, blinding, and any other potential risk of bias.
Treatment details: name of different therapies, dosage, frequency, session, and duration.
Outcome measurements: first, secondary, safety, and any other outcome instruments or tools.

#### 2.5.3 Risk of bias assessment

The Cochrane Risk of Bias Tool will be utilized for assessing the methodological quality for each qualified study through 7 different items. Each item will be further judged as low risk of bias, unclear risk of bias, or high risk of bias. Two researchers independently assess the methodological quality for each included study. Any disagreements will be settled down by discussion with a third researcher.

#### 2.5.4 Treatment effect measurement

For discontinuous outcome data, risk ratio with 95% confidence intervals (CIs) will be used to describe. For continuous outcome data, mean difference or standardized mean difference with 95% CIs will be utilized to expression.

#### 2.5.5 Dealing with missing data

Any missing data will be contacted with primary authors by using email. If we can not achieve those data, the available data will be analyzed only. However, we will discuss its impacts as limitation.

#### 2.5.6 Assessment of heterogeneity

*I*^2^ test will be performed to measure the heterogeneity. A fixed-effect model will be used if heterogeneity will be considered as low with *I*^2^ ≤50%. Otherwise, a random-effect will be applied if significant heterogeneity will be considered with *I*^2^ >50%.

#### 2.5.7 Assessment of reporting biases

If sufficient qualified studies (more than 10 studies) are included in this systematic review, we will also carry out the funnel plot and Egger regression test to check any publication bias.

#### 2.5.8 Data synthesis

RevMan 5.3 software will be utilized for data pooling and meta-analysis performance. Whenever there is low heterogeneity, a fixed-effect model will be applied, and meta-analysis will be performed. Otherwise, if there is significant heterogeneity, a random-effect model will be used to pool the data. Meanwhile, subgroup analysis will be performed. Meta-analysis will be carried out if low heterogeneity is identified after subgroup analysis. Otherwise, we will not perform meta-analysis, and narrative summary descriptions will be reported.

#### 2.5.9 Subgroup analysis

Subgroup analysis will be performed to investigate any possible reasons that may cause significant heterogeneity in accordance with the different interventions, controls, and outcome instruments.

#### 2.5.10 Sensitivity analysis

Where appropriate, sensitivity analysis will also be operated to check the robust of the combined outcome results by eliminating the impact of low methodological quality studies.

## 3 Discussion

This systematic review will first assess the efficacy and safety of CS for the treatment of patients with NSCLC. The results of this systematic review will present a summary of the most present evidence on the efficacy and safety of CS for NSCLC. Its findings may provide helpful evidence either for clinical practice or for future studies.

## Author contributions

**Conceptualization:** Lei Han, Zhou-Xia Wei, Ai-Ying Jiang.

**Data curation:** Lei Han, Yu-Feng Lv, Ai-Ying Jiang.

**Formal analysis:** Lei Han, Yu-Feng Lv.

**Funding acquisition:** Zhou-Xia Wei.

**Investigation:** Ai-Ying Jiang.

**Methodology:** Lei Han, Zhou-Xia Wei, Yu-Feng Lv.

**Project administration:** Ai-Ying Jiang.

**Resources:** Lei Han, Zhou-Xia Wei, Yu-Feng Lv.

**Software:** Lei Han, Zhou-Xia Wei, Yu-Feng Lv.

**Supervision:** Ai-Ying Jiang.

**Validation:** Lei Han, Zhou-Xia Wei, Yu-Feng Lv, Ai-Ying Jiang.

**Visualization:** Zhou-Xia Wei.

**Writing – original draft:** Lei Han, Zhou-Xia Wei, Ai-Ying Jiang.

**Writing – review & editing:** lei Han, Zhou-Xia Wei, Yu-Feng Lv, Ai-Ying Jiang.
